# The Clinical Effectiveness of Platelet-Rich Fibrin in Post-Extraction Healing: A Systematic Review of Randomized Controlled Trials

**DOI:** 10.3390/medicina62091672

**Published:** 2026-08-31

**Authors:** Liene Janaite, Irina Vasilcenko, Ingrida Cema, Ieva Bagante

**Affiliations:** 1 Faculty of Dentistry, Riga Stradins University, LV-1007 Riga, Latvia; liene.janaite@rsu.edu.lv; 2 Department of Oral and Maxillo-Facial Surgery and Oral Medicine, Riga Stradins University, LV-1007 Riga, Latvia; ingrida.cema@rsu.lv (I.C.); ieva.bagante@rsu.lv (I.B.); 3 Centre of Oral and Maxillo-Facial Surgery, P. Stradins Clinical University Hospital, LV-1002 Riga, Latvia

**Keywords:** platelet-rich fibrin, leukocyte–platelet-rich fibrin, tooth extraction, socket healing, alveolar osteitis, third molar surgery, bone regeneration, wound healing

## Abstract

*Background and Objectives*: Platelet-rich fibrin (PRF) has been widely investigated as a biological material to enhance healing after tooth extraction; however, its clinical effectiveness remains uncertain. This systematic review evaluates the available evidence on its effectiveness in post-extraction socket management. *Materials and Methods*: A literature search was conducted across eight electronic databases for English-language studies published between 2015 and 2025. Only randomized controlled trials, including parallel-group randomized trials and randomized split-mouth trials, involving PRF with a control group were considered. The methodological quality of the included studies was assessed using the Cochrane Risk of Bias tool (RoB 1). Due to substantial clinical heterogeneity in PRF preparation protocols, outcome measures, and follow-up periods, a narrative synthesis was performed instead of a meta-analysis. *Results*: A total of 174 records were identified, of which 9 studies met the inclusion criteria. The methodological quality of the studies included was evaluated to assess the risk of bias, in accordance with the recommendations of the Cochrane Handbook for Systematic Reviews of Interventions. Overall, 394 patients underwent 817 tooth extractions: 414 sockets were treated with different types of PRF, 251 were left to heal after suturing, and 143 were filled with a hemostatic sponge prior to suturing. *Conclusions*: PRF demonstrated superior outcomes in soft tissue healing and radiographic new bone density, although no statistically significant differences were observed in other parameters. Therefore, the available evidence does not allow us to draw definitive conclusions regarding the clinical effectiveness of PRF for socket filling after tooth extraction. The literature indicates that it may promote soft and hard tissue healing and reduce pain, swelling, and the risk of alveolar osteitis; however, evidence regarding its therapeutic effect on alveolar socket healing remains contradictory.

## 1. Introduction

Autologous platelet concentrates (APCs) have, in recent years, emerged as one of the most important biomaterials in regenerative dentistry. They are widely used in various clinical settings, including sinus lift procedures, peri-implantitis therapy, the management of medication-related osteonecrosis of the jaw, the filling of alveolar bone defects, and the preservation of the alveolar ridge following tooth extraction [1,2]. Following tooth extraction, intensive remodeling of the alveolar socket occurs, resulting in an average bone loss of 25–30% within the first two months, which may reach up to 40–60% of the initial alveolar bone volume within 2–3 years. These changes may compromise subsequent implant and prosthetic treatment outcomes and prognosis [3]. To mitigate bone loss and promote both soft and hard tissue healing, several generations of APCs have been developed [2]. Among these, platelet-rich fibrin (PRF) has gained particularly widespread application in contemporary practice.

Platelet-rich fibrin (PRF) comprises several preparation protocols, including leukocyte–platelet-rich fibrin (L-PRF), advanced platelet-rich fibrin (A-PRF), advanced platelet-rich fibrin plus (A-PRF+), and injectable platelet-rich fibrin (I-PRF). Throughout this review, the term “PRF” is used as a general designation for platelet-rich fibrin preparations unless a specific subtype is explicitly indicated.

Studies have reported that PRF preparations may reduce postoperative symptoms, including pain, facial swelling, trismus, and secondary bleeding [4,5]. PRF contains a high concentration of platelets, leukocytes, and a broad spectrum of growth factors that stimulate angiogenesis, osteoblastic activity, and soft tissue healing. A key advantage of PRF is that it is obtained without the use of anticoagulants, allowing for natural fibrin polymerization and the formation of a biologically compatible matrix suitable for socket filling and the promotion of bone regeneration [3,6].

Despite its promising biological potential demonstrated in vitro studies, clinical evidence regarding the effectiveness of PRF remains limited, inconsistent, and of low methodological quality [7,8,9,10]. Some studies fail to confirm its beneficial effects, whereas others report clinically significant improvements, indicating substantial heterogeneity in the available literature. These inconsistencies highlight the need for further high-quality studies to evaluate the clinical efficacy of PRF. Moreover, although the use of PRF in dentistry is increasing, clinical recommendations remain incomplete and are based on limited evidence. A systematic analysis of the literature would enable a more objective assessment of the true effectiveness of PRF by synthesizing data from various studies, thereby providing a clearer understanding of its role in routine clinical practice. Furthermore, because PRF preparation protocols have evolved considerably over time, previous systematic reviews included studies performed using heterogeneous preparation protocols that may not fully reflect contemporary clinical practice. In addition, several randomized controlled trials, including parallel-group randomized trials and randomized split-mouth trials, published after the most recent systematic reviews were not included in earlier evidence syntheses, warranting an updated review of the available literature. The aim of this study was to systematically evaluate the available evidence regarding the use of platelet-rich fibrin (PRF) following tooth extraction and to assess its effects on postoperative complications and tissue healing compared with conventional management without PRF.

## 2. Materials and Methods

### 2.1. Search Strategy

**Figure 1:**
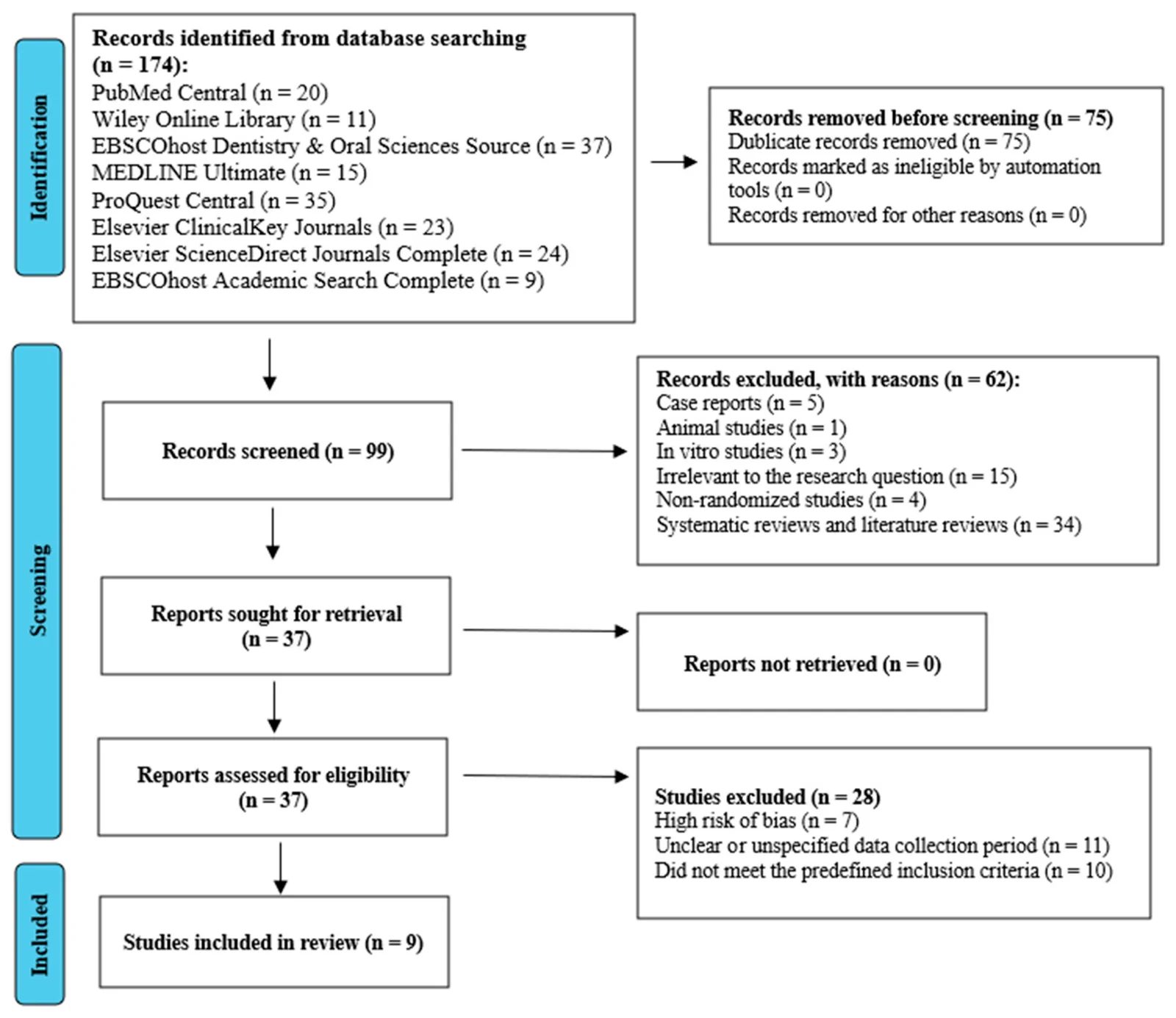
PRISMA (Preferred Reporting Items for Systematic Reviews and Meta-Analyses) flow diagram for study selection.

This systematic review was conducted and reported in accordance with the PRISMA 2020 (Preferred Reporting Items for Systematic Reviews and Meta-Analyses) guidelines [11], and the completed PRISMA 2020 checklist is provided in the Supplementary Materials (Table S2). The study selection process is illustrated in Figure 1. This study design did not require approval from an ethics committee or the obtaining of written informed consent from patients. The review protocol was not prospectively registered, and no formal review protocol was prepared.

The development of this study was based on research articles retrieved from the following electronic databases: Wiley Online Library, EBSCOhost Dentistry & Oral Sciences Source, MEDLINE Ultimate, ProQuest Central, Elsevier ClinicalKey Journals, Elsevier ScienceDirect Journals Complete, EBSCOhost Academic Search Complete, and PubMed Central. 

The electronic search strategy was developed using free-text keywords related to platelet-rich fibrin and tooth extraction. Boolean operators (“AND” and “OR”) were used to combine the search terms. The primary search string was as follows: (“platelet-rich fibrin” OR PRF OR “leukocyte platelet-rich fibrin” OR L-PRF OR “advanced platelet-rich fibrin” OR A-PRF OR “injectable platelet-rich fibrin” OR I-PRF) AND (“tooth extraction” OR “dental extraction” OR exodontia OR “extraction socket” OR “socket healing” OR “alveolar socket”) AND (“wound healing” OR “bone regeneration” OR “alveolar ridge preservation” OR “alveolar osteitis” OR “dry socket” OR pain OR swelling OR trismus). Filters were applied to include studies published in English between January 2015 and August 2025 involving human participants. The search was limited to studies published between 2015 and 2025 to ensure methodological and clinical comparability. Earlier studies were excluded because advances in PRF preparation protocols, centrifugation parameters, and clinical application techniques have substantially changed the characteristics of PRF products used in contemporary practice. The final electronic search was conducted on 18 August 2025.

The search strategy was structured according to the PICOS framework, encompassing (P) population, (I) intervention, (C) comparison, (O) outcomes, and (S) study design. Based on this framework, the research question was formulated using the following criteria: (P) patients requiring tooth extraction; (I) socket filling with platelet-rich fibrin (PRF) following tooth extraction; (C) suturing or the use of other biomaterials without PRF; (O) the incidence of postoperative complications (e.g., alveolar osteitis), pain intensity, and rate of tissue healing; (S) randomized controlled trials. 

### 2.2. Inclusion and Exclusion Criteria

Study selection included only articles published in English that reported on the use of platelet-rich fibrin (PRF) following tooth extraction in accordance with the predefined search strategy. Eligibility was restricted to randomized controlled trials involving human participants, including both parallel-group randomized trials and randomized split-mouth trials. Only full-text articles comparing PRF with a control group were eligible for inclusion.

Publications that did not meet the inclusion criteria were excluded. Excluded studies comprised all animal and in vitro studies, as well as studies involving immediate implant placement and non-randomized designs. In addition, systematic reviews, meta-analyses, and narrative literature reviews were excluded, along with studies that did not meet the predefined methodological quality criteria. Studies were required to clearly report the data collection period to ensure that the PRF preparation protocols evaluated reflected contemporary clinical practice. Because PRF preparation protocols have undergone substantial changes over time, studies in which the data collection period was not reported could not be reliably linked to the protocols currently used in clinical practice. Therefore, only studies including data collected from January 2015 onward were considered. Studies for which the data collection period could not be established were excluded.

### 2.3. Study Selection and Data Extraction

Title and abstract screening were initially performed by one reviewer (L.J.). Potentially eligible studies were subsequently discussed with the co-authors, and decisions regarding full-text assessment and final study inclusion were reached by consensus. Data extraction and risk of bias assessment were performed by L.J. and I.B. Any disagreements regarding study eligibility, extracted data, or risk of bias assessment were resolved through discussion among the authors until consensus was achieved. After the removal of 75 duplicates, the titles and abstracts of 99 records were screened. Of these, 62 records were excluded because they did not meet the predefined inclusion and exclusion criteria. The full texts of the remaining 37 articles were retrieved and assessed for eligibility. Following full-text assessment, 28 articles were excluded for documented reasons. Of these, seven studies [12,13,14,15,16,17,18] were excluded from the final qualitative synthesis because they exceeded the predefined risk-of-bias threshold described in Section 2.4 (see articles excluded in Table S1). Nine studies were included in the final qualitative synthesis.

Data from the 9 included studies were extracted and recorded in an Excel (Microsoft) spreadsheet. The following information was collected for each study: author and year of publication, study design, duration of follow-up, number of participants, number of extracted teeth, type of intervention, assessment methods, and outcomes.

### 2.4. Quality Assessment of Included Studies

The methodological quality of the included studies was assessed using the Cochrane Risk of Bias tool for randomized controlled trials (RoB 1), as described in the Cochrane Handbook for Systematic Reviews of Interventions. The following domains were analyzed: random sequence generation; allocation concealment; blinding of participants and personnel; blinding of outcome assessment; incomplete outcome data; and selective outcome reporting. Although no formal review protocol was prospectively registered, the methodological eligibility criteria, including the predefined risk of bias threshold, were established before study selection and data extraction commenced. Randomized controlled trials with more than two RoB 1 domains judged as either high risk or unclear risk of bias were considered methodologically inadequate and were excluded from the final qualitative synthesis. These criteria were applied consistently throughout the review.

## 3. Results

A total of 174 records were identified across eight databases. After the removal of 75 duplicates, 99 records underwent title and abstract screening. Of these, 62 records were excluded because they did not meet the predefined eligibility criteria. The full texts of 37 articles were assessed for eligibility. Of these, 28 studies were excluded because they did not meet the predefined eligibility criteria. Specifically, 7 studies [12,13,14,15,16,17,18] were excluded from the qualitative synthesis because they presented a high risk of bias in more than two RoB 1 domains (the domain-level risk-of-bias assessments and reasons for methodological exclusion of these studies are provided in Table S1 and summarized in Figure S1), 11 did not report or clearly specify the data collection period, and 10 did not meet the remaining predefined inclusion criteria. Nine studies were included in the final qualitative synthesis (Figure 1).

Among the included studies, five employed a split-mouth design and four used a controlled parallel-group design. All included studies were randomized and prospective. A total of 394 patients underwent extractions of 817 teeth, including 20 third molars without osteotomy, 239 third molars requiring flap and osteotomy, 52 molars and premolars without osteotomy, 98 anterior teeth, and 408 teeth that were not specifically classified but were reported as non-adjacent and extracted without flap or osteotomy.

Overall, 414 sockets were filled with different types of platelet-rich fibrin (PRF), 251 sockets were sutured and left to heal spontaneously, and 143 sockets were filled with a hemostatic sponge prior to suturing (including 20 cases in which it was used in combination with PRF) (Table 1).

### 3.1. Risk of Bias Assessment

**Figure 2:**
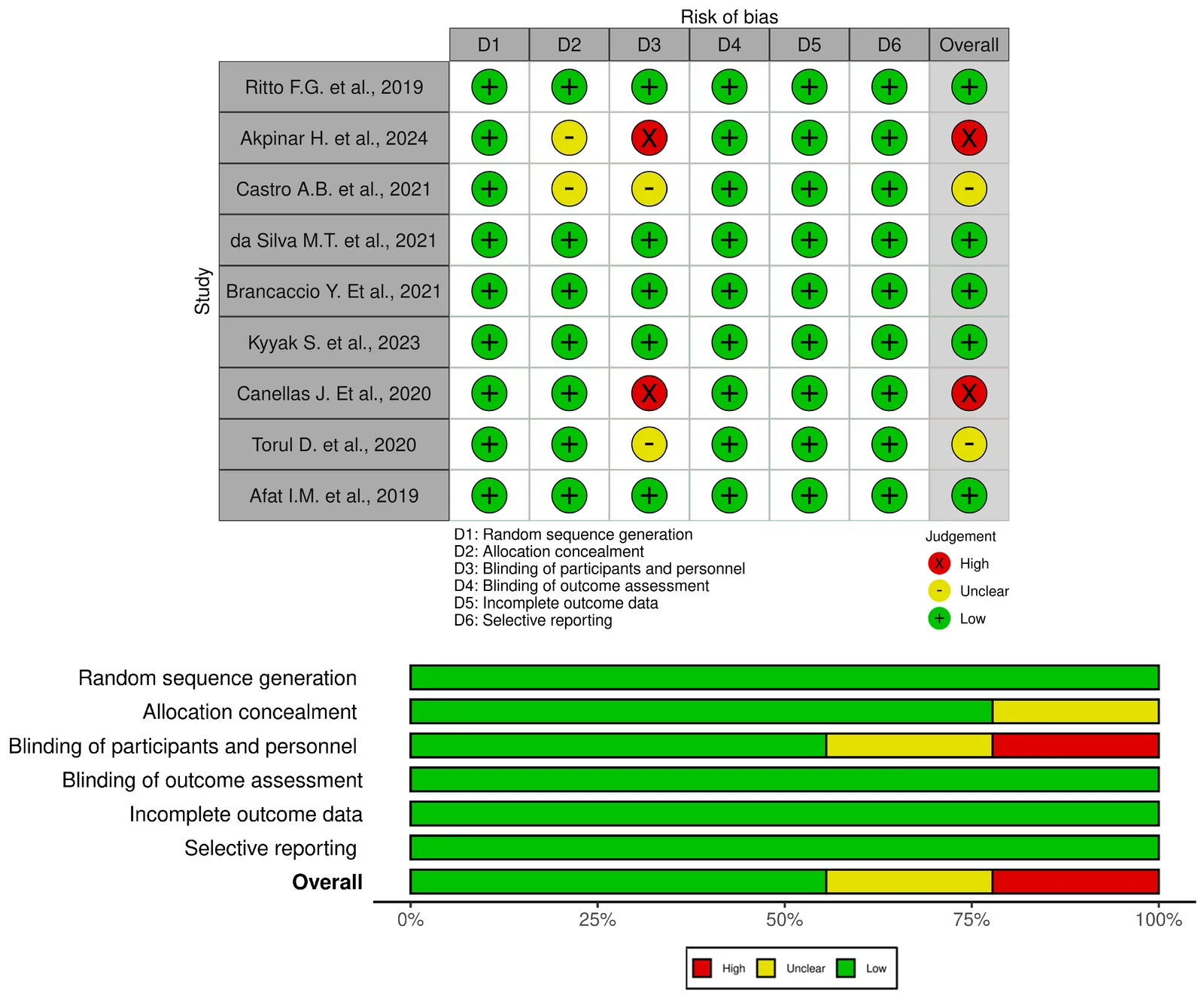
A summary of the risk of bias of the included studies [7,9,19,20,21,22,23,24,25].

All nine studies adequately described the method of random sequence generation using either computer-generated randomization or coin-toss methods (Figure 2).

Allocation concealment was adequately described in seven studies. In four studies, pre-generated allocation codes were placed in sequentially numbered, opaque, sealed envelopes that were opened only after tooth extraction, thereby preventing the surgeon from influencing treatment allocation [7,9,22,23]. In the remaining three studies, allocation concealment was achieved using other methods, including concealing the allocation sequence until participants were enrolled and assigned to the intervention or other procedures preventing foreknowledge of treatment allocation [21,24,25]. Allocation concealment was insufficiently described in the remaining two studies [19,20].

Regarding blinding of participants and personnel, two of the nine studies provided a detailed description of how participant blinding was achieved. In the study by Ritto et al. (2019) [9], patients were provided with dark glasses, while da Silva et al. (2021) [21] simulated PRF application on the control side to prevent participants from identifying the intervention site. These studies were therefore considered to have a low risk of bias. In seven studies, the authors reported that participants remained unaware of the allocated treatment until completion of the study [7,9,20,21,22,24,25]. In contrast, in the studies by Akpınar et al. (2024) and Canellas et al. (2020), participants were able to visually identify the side receiving PRF during the surgical procedure, resulting in a high risk of bias for participant blinding [19,23].

Regarding blinding of personnel, Ritto et al. (2019) and Kyyak et al. (2023) reported that the surgical procedures were performed by a clinician who was blinded to treatment allocation, whereas a second operator, who was not involved in tooth extraction or postoperative outcome assessment, was responsible for PRF placement and wound closure or suturing of the control site [9,22]. These procedures minimized the risk of performance bias associated with the operating surgeon. In the remaining studies, the same surgeon performed both tooth extraction and PRF application and was therefore aware of the allocated intervention.

With respect to blinding of outcome assessment, all nine studies were considered to have a low risk of bias. Outcome measurements were performed by independent examiners who were not involved in the treatment procedures and were unaware of treatment allocation. Furthermore, patient data were coded before analysis, ensuring that outcome assessment was conducted without knowledge of the allocated intervention.

Regarding incomplete outcome data and selective reporting, all nine studies were judged to have a low risk of bias. All studies reported complete outcome data, and no evidence of selective reporting was identified.

### 3.2. Postoperative Pain

Pain intensity was assessed in the studies reporting pain outcomes using a visual analog scale (VAS) ranging from 0 (no pain) to 10 (worst imaginable pain). Four studies reported outcomes related to pain intensity. In three of these studies, no statistically significant differences in VAS scores were observed between groups at any time point, although a slight reduction in pain was noted in the PRF group [9,24,25]. In contrast, one study reported significantly lower mean VAS scores at sites treated with L-PRF on all evaluated days; however, when pain was analyzed using the time-weighted summed pain intensity difference (SPID), no statistically significant differences were found between the L-PRF and control groups. Pain was evaluated at 24 h, 48 h, 72 h, and 7 days postoperatively in all studies [21].

### 3.3. Use of Nonsteroidal Anti-Inflammatory Drugs

In the study by Akpınar et al. (2024), it was reported that the placement of PRF in the extraction socket significantly reduced the use of nonsteroidal anti-inflammatory drugs (NSAIDs) during the first 48 h of the postoperative period; however, no statistically significant differences in analgesic consumption were observed between groups in the subsequent days [19]. In contrast, two other studies reported no statistically significant differences in analgesic intake across different time points [21,24]. 

### 3.4. Swelling and Trismus

In the study by Akpınar et al. (2024) [19], postoperative swelling was evaluated, and a statistically significant reduction was observed in measurements taken from the lateral canthus to the angle of the mandible. However, no statistically significant differences were found between groups in tragus–pogonion measurements on either the second or seventh postoperative day [19]. Similarly, Torul et al. (2020) reported no statistically significant differences in swelling between the PRF and control groups [24].

These two studies were also the only ones that assessed trismus. Akpınar et al. (2024) [19] reported a statistically significant difference between the PRF and control groups on postoperative day 2, whereas no significant difference was observed on day 7. In contrast, Torul et al. (2020) found no statistically significant differences between the groups at any time point [19,24].

### 3.5. Alveolar Bone Loss and Bone Regeneration

Among the nine included studies, two evaluated alveolar bone loss and new bone density using computed tomography immediately after extraction and at 3 months. One study reported no significant differences in alveolar bone loss or in vertical/horizontal or buccal/palatal bone resorption between groups, whereas the other indicated that PRF reduced alveolar bone loss [9,20].

Both studies reported a statistically significant increase in radiographic bone density within the extraction socket after 3 months compared with the control group. However, it should be noted that in the study by Castro et al. (2021), a significantly greater bone volume was observed only in the A-PRF+ group, but not in the L-PRF group, when compared with controls [20].

In two studies, patients opted for implant placement following tooth extraction. During implant insertion, bone biopsies were obtained from each socket using a 2 mm diameter trephine bur. Castro et al. (2021) [20] reported that PRF application did not result in statistically significant changes in bone structure compared with the control group. In contrast, Canellas et al. (2020) [23] demonstrated that PRF preparations significantly enhanced new bone formation (55.96 ± 11.97%) after 3 months compared with the control group (39.69 ± 11.13%), although no significant differences were observed in the proportion of mature versus immature bone. Overall, no statistically significant differences between PRF and control groups were identified in this regard [20,23].

### 3.6. Soft Tissue Healing

Four studies evaluated soft tissue healing. Among these, Ritto et al. (2019) [9] reported no differences compared with the control group, suggesting that PRF placement in the extraction socket does not influence soft tissue healing. In contrast, da Silva et al. (2021) and Afat et al. (2019) demonstrated that PRF preparations significantly accelerated and improved soft tissue healing compared with the control group; however, no statistically significant differences were observed between the PRF group and the hemostatic sponge group [9,21,25].

Similarly, Brancaccio et al. (2021) [7] reported statistically significant differences in healing rate when comparing sockets treated with L-PRF to both the control group and the hemostatic sponge group. A-PRF+ demonstrated superior healing compared specifically with the hemostatic sponge; however, a significantly lower risk of incomplete healing was observed only in the L-PRF group [7].

### 3.7. Risk of Postoperative Bleeding

Of the nine included studies, three reported outcomes related to bleeding (one involving patients using factor Xa inhibitors, one involving patients on anticoagulants, and one involving patients not taking medication). In the study by Kyyak et al. (2023) [22], no statistically significant differences were observed compared with the control group (treated with a hemostatic gelatin sponge), and no cases of postoperative bleeding were reported. However, a correlation was identified between bleeding duration and the location of the extracted teeth, with prolonged bleeding observed in posterior regions [22].

In contrast, Brancaccio et al. (2021) reported a statistically significant reduction in bleeding when comparing both A-PRF+ and L-PRF groups with the control group, as well as when comparing A-PRF+ with the hemostatic sponge group; no significant differences were observed in the remaining comparisons [7]. Afat et al. (2019) reported no cases of prolonged or delayed postoperative bleeding in any of the groups [25].

### 3.8. Alveolar Osteitis and Other Complications

In three of the nine studies, no adverse effects or postoperative complications were reported. Afat et al. (2019) [25] noted a single case of alveolar osteitis in the control group, which was not statistically significant. The remaining five studies did not provide information regarding additional complications [25].

## 4. Discussion

In this systematic review, only randomized controlled trials, including parallel-group randomized trials and randomized split-mouth trials, meeting the predefined eligibility criteria and considered to be of sufficient methodological quality were included. Although many in vitro, in vivo, and clinical studies addressing the selected topic are available, the number of high-quality systematic reviews remains limited. Differences were observed between the present review and previously published systematic reviews regarding the assessment of methodological quality and risk of bias. These differences may have influenced the interpretation of the available evidence regarding the effectiveness of PRF.

In the systematic review by Bao et al. (2021), ten studies were analyzed, of which eight were classified as having a low risk of bias and two as unclear [26]. However, application of the predefined risk of bias assessment criteria used in the present review resulted in six studies being classified as having a high risk of bias. Moreover, one study lacked a control group. According to the predefined methodological criteria applied in the present review, only three studies were considered to have a low overall risk of bias. Despite this, the review reported no statistically significant differences between PRF and control groups across the evaluated outcomes. Notably, several studies comparing different PRF protocols did not report randomization procedures or adequate blinding of participants, surgeons, and outcome assessors. These differences in risk of bias assessment may contribute to discrepancies in the interpretation of study quality and the reported clinical findings.

Similarly, in the systematic review by Zhu et al. (2021), nineteen studies were included and initially classified as having either a low or unclear risk of bias [10]. However, application of the predefined risk of bias assessment criteria used in the present review resulted in five studies being classified as having a low risk of bias, five demonstrated a moderate or unclear risk, and nine exhibited a high risk of bias due to the absence of essential methodological information, such as data collection timing, randomization procedures, group allocation, and blinding of participants, clinicians, and outcome assessors. The results of this review indicated a statistically significant reduction in the incidence of alveolar osteitis and pain, as well as a significant decrease in swelling and nonsteroidal anti-inflammatory drug consumption in the PRF group compared with controls on postoperative days 1, 3, and 7; however, no significant differences were observed in trismus. It is important to note that Ye et al. (2024) [27] analyzed the same studies as Zhu et al. (2021) [10], while adding 16 additional studies, and concluded that all included studies were of high or moderate quality. Nevertheless, when the predefined methodological criteria applied in the present review were used, only six of these additional studies fulfilled the criteria for high or moderate methodological quality [10,27].

In the present review, four studies included in previously published systematic reviews were also incorporated: Ritto et al. (2019), which was included in all reviewed articles, as well as Afat et al. (2019), Torul et al. (2020), and da Silva et al. (2021), which were included in at least one review [9,21,24,25].

According to Ye et al. (2024), any tooth extraction may be considered an indication for PRF application to reduce the risk of non-infectious postoperative complications [27]. In contrast, in the present systematic review—restricted to randomized controlled trials, including parallel-group randomized trials and randomized split-mouth trials meeting the predefined methodological eligibility criteria—almost half of the included studies reported no postoperative complications, even after a follow-up period of up to 3 months, while the remaining studies did not provide data on additional complications.

An important methodological observation of the present review is the considerable variability in PRF preparation protocols and their application in clinical studies. The included studies reported heterogeneous centrifugation protocols. For example, although six studies described the use of L-PRF, centrifugation parameters varied, ranging from 3000 rpm for 10 min in one study to 2700 rpm for 12 min in others [7,9,20,21,23,25]. Similarly, A-PRF preparation protocols also differed, ranging from 1200–1300 rpm for 8 min to 1300 rpm for 14 min [22,24].

This variability is further supported by studies not included in the present review. For instance, Asoka et al. (2022) reported centrifugation at 3000 rpm for 12 min, while Malhotra et al. (2020) and Alzahrani et al. (2017) used 3000 rpm for 10 min, and Asif et al. (2023) reported 3500 rpm for 10 min [18,28,29,30]. Similarly, A-PRF protocols ranged from 1500 rpm for 14 min to 1300 rpm for 8 min [17,31].

Another important limitation is that most studies did not report the time interval between blood collection and centrifugation, making it difficult to objectively assess the quality of the obtained PRF. Several authors have emphasized that PRF quality is highly dependent on operator performance and procedural precision. For example, Borie et al. (2015) highlighted that PRF quality depends on the timing of blood collection and immediate centrifugation [32]. Pavlovic et al. (2021) recommended that centrifugation should begin within 2 min and 30 s, while Quirynen et al. (2025) suggested an even stricter interval of approximately 1 min [33,34]. Exceeding this time frame may result in uncontrolled fibrin polymerization, rendering the material clinically unsuitable. Miron et al. (2019) demonstrated that even a short delay of 60–90 s can alter the structure of the PRF membrane, while a delay of 120 s may reduce membrane size by approximately 23% [35]. Therefore, operator experience and technical precision represent critical factors influencing PRF quality, and both methodological and human factors must be considered when interpreting clinical outcomes.

An additional factor that may influence the effectiveness of platelet-rich fibrin (PRF) is the use of nonsteroidal anti-inflammatory drugs (NSAIDs). The biological activity of PRF depends on platelet activation and the subsequent release of growth factors involved in tissue regeneration and wound healing. Evidence suggests that NSAIDs may interfere with platelet function by inhibiting cyclooxygenase activity and reducing thromboxane A2 production, thereby impairing platelet activation and aggregation. As a result, the regenerative potential of platelet concentrates may be altered [36]. Previous investigations have demonstrated that antiplatelet and NSAID medications can affect platelet responsiveness and modify the biological characteristics of autologous platelet preparations, potentially influencing their clinical performance. Despite the relevance of this factor, none of the studies included in the present review reported whether participants used NSAIDs before blood collection and PRF preparation. Consequently, the potential impact of medication use on PRF quality and treatment outcomes cannot be excluded and should be considered in the design and interpretation of future clinical trials [36,37].

Despite the widespread use of PRF following tooth extraction, there is still no consensus in the literature regarding the optimal and clinically most appropriate application protocol. To reliably assess the clinical effectiveness of PRF and to define its role in enhancing healing processes, further high-quality randomized controlled trials are required.

This review has several limitations. The review protocol was not prospectively registered, and no formal GRADE assessment of the certainty of the evidence was performed. In addition, the exclusion of studies according to an author-defined risk of bias threshold may have reduced the comprehensiveness of the evidence base. Considerable clinical and methodological heterogeneity, including differences in PRF preparation protocols, comparator interventions, outcome measures, and follow-up periods, precluded quantitative meta-analysis and limits the generalizability of the findings.

## 5. Conclusions

Several types of platelet-rich fibrin (PRF) exist, including L-PRF, A-PRF, A-PRF+ and I-PRF, which differ in their cellular composition, concentration of growth factors, and preparation protocols. The methods of PRF preparation and their resulting quality are influenced by multiple factors, particularly operator expertise. Currently, no standardized protocol for PRF preparation and clinical application following tooth extraction has been established. Standardization of PRF preparation protocols is essential to improve the comparability of future clinical studies and to facilitate evidence-based clinical recommendations. Variations in preparation protocols significantly affect the composition of the final product, allowing for modulation of specific biologically active components within the PRF matrix.

The literature suggests that PRF may promote both soft and hard tissue healing, and reduce pain, swelling, and the risk of alveolar osteitis. However, the available clinical evidence remains limited and heterogeneous, and its therapeutic effectiveness in socket preservation after tooth extraction cannot yet be established with certainty. L-PRF and A-PRF appear to demonstrate more favorable outcomes in terms of soft tissue healing and radiographic bone density, whereas no statistically significant differences are consistently observed for other clinical parameters.

No serious adverse effects were reported in the studies that explicitly assessed postoperative complications. The findings of the present systematic review suggest potential clinical benefits of PRF; however, they should be interpreted with caution because of methodological limitations and heterogeneity among the included studies. Further well-designed, adequately powered randomized controlled trials using standardized PRF preparation protocols are required to establish its clinical effectiveness and determine the optimal protocol for routine clinical practice.

## Figures and Tables

**Table 1:** Characteristics of included studies.

Author, Year	Data Collection Period	Type of Study	Follow-Up	Number of Participants (*n*)	Number of Extracted Teeth (*n*)	Intervention Type	Measurement Method	Centrifugation Protocol
Ritto F.G. et al. (2019) [9]	2016	RCT, Split mouth	3 months	17	34	L-PRF\control	CBCT, VAS	2700 rpm, 12 min
Akpınar H. et al. (2024) [19]	2022	RCT, Split mouth	7 days	35	70	I-PRF/ control	VAS, Swelling, postoperative symptom severity scale	700 rpm, 3 min
Castro A.B. et al. (2021) [20]	2015–2018	RCT, Split mouth	3 months	20	60	L-PRF/ A-PRF+/ control	CBCT, 2D analysis, biopsy analysis, histology, morphometric analysis	2700 rpm, 12 min/1300 rpm, 8 min
da Silva M.T. et al. (2021) [21]	2019	RCT, Split mouth	2 weeks	20	20	L-PRF/ control	VAS, growth factor levels, tissue healing	2700 rpm, 12 min
Brancaccio Y. et al. (2021) [7]	2017–2019	RCT	2 weeks	102	408	L-PRF/ A-PRF+/hemostatic sponge/control	Bleeding, tissue healing	2700 rpm, 12 min/1300 rpm, 8 min
Kyyak S. et al. (2023) [22]	2022	RCT, Split mouth	1 week	21	42	A-PRF/ hemostatic sponge	Bleeding	1200 rpm, 8 min
Canellas J. et al. (2020) [23]	2018	RCT	3 months	44	44	L-PRF/ control	CBCT, histology	2700 rpm, 12 min
Torul D. et al. (2020) [24]	2018–2019	RCT	1 week	75	75	A-PRF/control/CGF	Swelling, VAS, trismus	1300 rpm, 14 min
Afat I.M. et al. (2019) [25]	2016	RCT	3 weeks	60	60	L-PRF/PRF + HA/control	Healing, bleeding, alveolitis	3000 rpm, 10 min

A-PRF+: Advanced platelet-rich fibrin plus; CBCT: Cone beam computed tomography; I-PRF: Injectable platelet-rich fibrin; L-PRF: Leukocyte-platelet-rich fibrin; PRF: Platelet-rich fibrin; RCT: Randomized Controlled Trial; PRF + HA: Platelet-rich fibrin (L-PRF) combined with hyaluronic acid sponge; VAS: Visual analog scale for pain intensity assessment (0—no pain, 10—worst imaginable pain).

## Data Availability

No new data were created or analyzed in this study. Data sharing is not applicable to this article, as the study was based exclusively on previously published literature.

## Supplementary Materials

The following supporting information can be downloaded at: https://www.mdpi.com/article/10.3390/medicina62091672/s1, Table S1: Studies excluded on the basis of the predefined risk of bias threshold together with their domain-level RoB 1 assessments; Figure S1: Risk-of-bias summary for studies excluded from the qualitative synthesis on the basis of the predefined RoB 1 threshold; Table S2: PRISMA 2020 checklist.

## Abbreviations

The following abbreviations are used in this manuscript:

A-PRF+	Advanced platelet-rich fibrin plus
APC	Autologous platelet concentrate
CBCT	Cone beam computed tomography
I-PRF	Injectable platelet-rich fibrin
L-PRF	Leukocyte-–platelet-rich fibrin
PRF	Platelet-rich fibrin
RCT	Randomized Controlled Trial
SPID	Sum of pain intensity differences over a defined time period, reflecting both the intensity and duration of pain
VAS	Visual analog scale for pain intensity assessment (0—no pain, 10—worst imaginable pain)
