# Supplementary material for: The Clinical Effectiveness of Platelet-Rich Fibrin in Post-Extraction Healing: A Systematic Review of Randomized Controlled Trials
Source: Medicina (Kaunas). 2026 Aug 31;62(9):1672. doi: 10.3390/medicina62091672 (PMC13609539; doi:10.3390/medicina62091672)
Supplement: Supplementary file 1 [file medicina-62-01672-s001.zip › Supplement Table S1 and Figure S1.pdf]

Clinical Effectiveness of Platelet-Rich Fibrin in Post-Extraction Healing: A Systematic Review of Randomized Controlled Trials

Liene Janaite , Irina Vasilcenko, Ingrida Cema and Ieva Bagante

Table S1: Studies excluded on the basis of the predefined risk-of-bias threshold together with their domain-level RoB 1 assessments.

**Author, year**	Random sequence generation	Allocation sequence concealment	Blinding of participants and personnel	Blinding of outcome assessment	Incomplete outcome data	Selective outcome reporting	
Alrayyes Y. et all (2022)	+	?	X	X	+	+	Alrayyes, Y., Aloraini, S., Alkhalaf, A., & Aljasser, R. (2022). Soft-Tissue Healing Assessment after Extraction and Socket Preservation Using Platelet-Rich Fibrin (PRF) in Smokers: A Single-Blinded, Randomized, Controlled Clinical Trial. Diagnostics (Basel), 12(10), 2403. https://doi.org/10.3390/diagnostics12102403
Talha J. et all (2024)	?	?	X	X	+	?	Talha Javed, Bushra Arain, Mowaffaq Abdullmomen Al Absi, Zunair Akbar Memon, Hafiz Mahmood, Aswa Ahmed, & Waqas Iqbal. (2024). Comparison of Platelet-rich fibrin with Zinc oxide eugenol for the pain management of Alveolar Osteitis. The Professional Medical Journal, 31(6), 972–976. https://doi.org/10.29309/TPMJ/2024.31.06.7565
Aliyev T. et all (2025)	+	+	X	?	+	?	Aliyev, T., Ulu, M., Rizaj, X., Şahin, O., Şimşek, F., & Davudov, M. (2025). Does varying platelet-rich fibrin centrifugation protocols enhance new bone formation in extraction site? Journal of Oral and Maxillofacial Surgery, 83(1), 62–69. https://doi.org/10.1016/j.joms.2024.09.012
Śmieszek-Wilczewska	+	?	X	?	+	?	Śmieszek-Wilczewska, J., Balicz, A., Morawiec, T., Al-Maawi, S., Heselich, A., Sader, R., … Ghanaati, S. (2024).

J. et all (2024)							Effectiveness of oroantral communication closure using solid platelet-rich fibrin compared to a conventional treatment approach: A randomized clinical trial. The Journal of Oral Implantology, 50(1), 3–8. https://doi.org/10.1563/aaid-joi-D-23-00108
Iqbal N. et all (2023)	+	X	X	+	X	?	Iqbal, N., Khalid, M. U., Janjua, O. S., Zafar, K. J., & Usama, M. M. (2023). Assessment of Dry Socket after Mandibular Third Molar Surgery Using Platelet-Rich Fibrin - A Prospective Clinical Study. Journal of the College of Physicians and Surgeons--Pakistan, 33(5), 504–508. https://doi.org/10.29271/jcpsp.2023.05.504
Starzyńska A. et all (2021)	+	X	X	X	+	?	Starzyńska, A., Kaczoruk-Wieremczuk, M., Lopez, M. A., Passarelli, P. C., & Adamska, P. (2021). The Growth Factors in Advanced Platelet-Rich Fibrin (A-PRF) Reduce Postoperative Complications after Mandibular Third Molar Odontectomy. International Journal of Environmental Research and Public Health, 18(24), 13343. https://doi.org/10.3390/ijerph182413343
Asif M. et all (2023)	+	X	X	+	+	?	Asif, M.; Ullah, A.; Mujtaba, H.; Umer, M.F.; Khurshid, Z. Comparative Study of Frequency of Alveolar Osteitis, with and Without Using Platelet-Rich Fibrin in Mandibular Third Molar Surgery. Int. J. Dent. 2023, 2023, 2256113

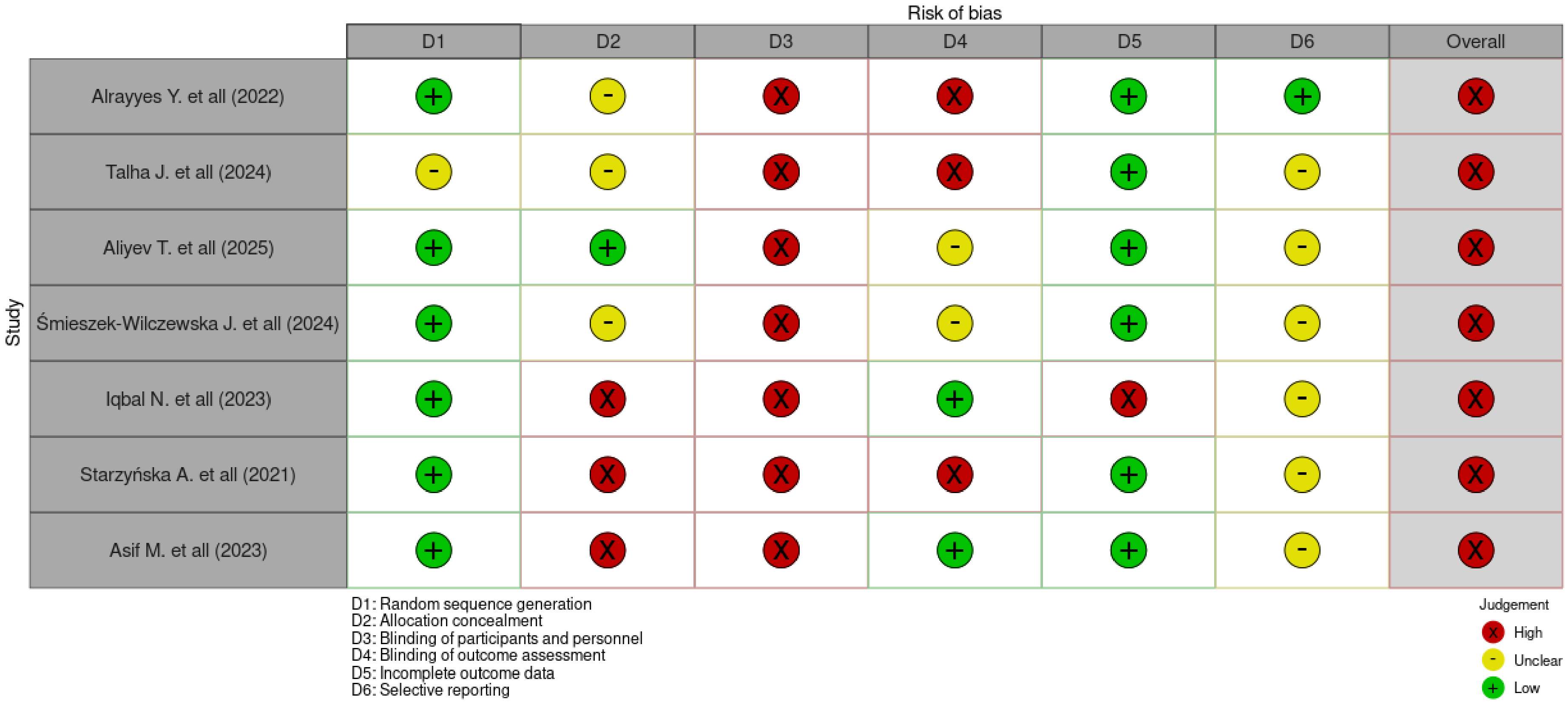
Risk of bias
D1
D2
D3
D4
D5
D6
Overall
Study
Alrayyes Y. et all (2022)
Talha J. et all (2024)
Aliyev T. et all (2025)
Śmieszek-Wilczewska J. et all (2024)
Iqbal N. et all (2023)
Starzyńska A. et all (2021)
Asif M. et all (2023)
D1: Random sequence generation
D2: Allocation concealment
D3: Blinding of participants and personnel
D4: Blinding of outcome assessment
D5: Incomplete outcome data
D6: Selective reporting
Judgement
High
Unclear
Low

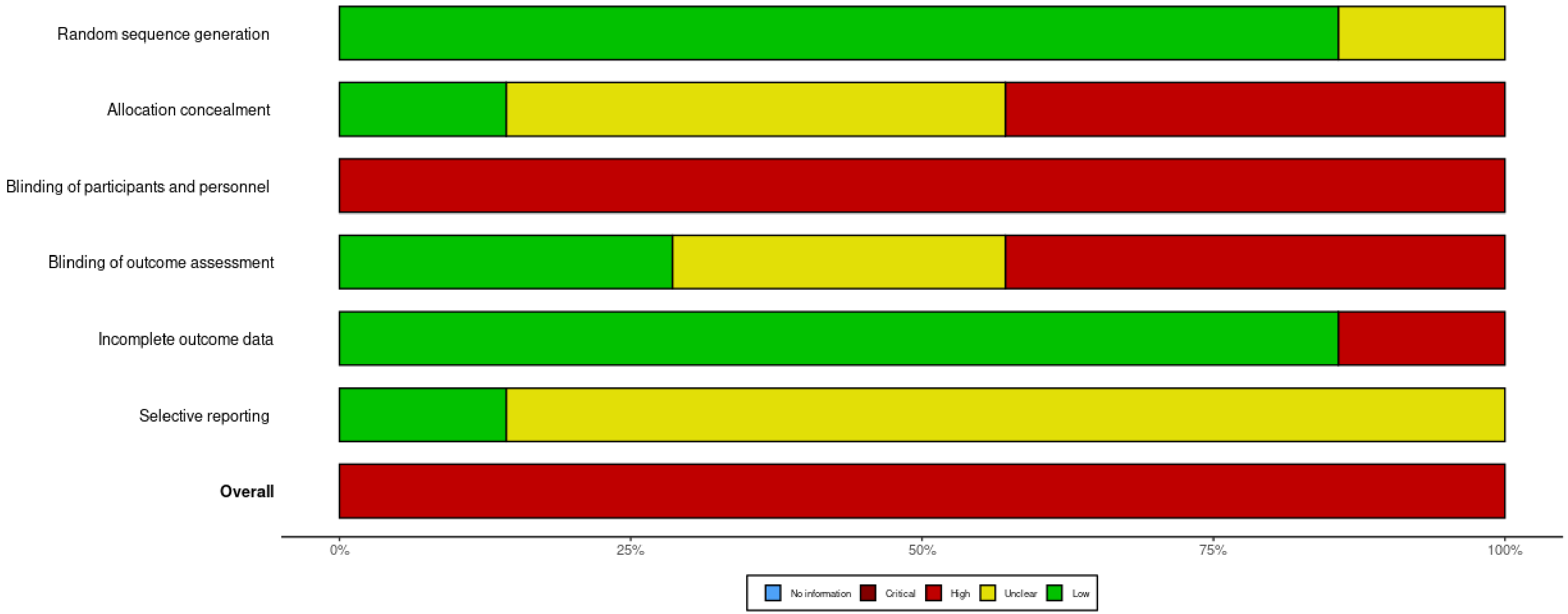

Random sequence generation
Allocation concealment
Blinding of participants and personnel
Blinding of outcome assessment
Incomplete outcome data
Selective reporting
Overall
0%
25%
50%
75%
100%
No information
Critical
High
Unclear
Low

Figure S1
