# Supplementary material for: The Clinical Effectiveness of Platelet-Rich Fibrin in Post-Extraction Healing: A Systematic Review of Randomized Controlled Trials
Source: Medicina (Kaunas). 2026 Aug 31;62(9):1672. doi: 10.3390/medicina62091672 (PMC13609539; doi:10.3390/medicina62091672)
Supplement: Supplementary file 1 [file medicina-62-01672-s001.zip › Table S2--PRISMA_2020_checklist_FINAL_formatted.pdf]

PRISMA 2020 Checklist

Section and Topic	Item #	Checklist item	Location where item is reported
**TITLE**			
Title	1	Identify the report as a systematic review.	Title, p. 1.
**ABSTRACT**			
Abstract	2	See the PRISMA 2020 for Abstracts checklist.	Abstract, p. 1.
**INTRODUCTION**			
Rationale	3	Describe the rationale for the review in the context of existing knowledge.	Introduction, p. 2.
Objectives	4	Provide an explicit statement of the objective(s) or question(s) the review addresses.	Introduction, p. 2.
**METHODS**			
Eligibility criteria	5	Specify the inclusion and exclusion criteria for the review and how studies were grouped for the syntheses.	Section 2.2, p. 4. Inclusion/exclusion criteria are reported
Information sources	6	Specify all databases, registers, websites, organisations, reference lists and other sources searched or consulted to identify studies. Specify the date when each source was last searched or consulted.	Section 2.1, pp. 3–4. Eight databases and the final search date (18 August 2025) are reported
Search strategy	7	Present the full search strategies for all databases, registers and websites, including any filters and limits used.	Section 2.1, p. 3-4. The primary search string, filters, language, date range, and human-participant limits are reported
Selection process	8	Specify the methods used to decide whether a study met the inclusion criteria of the review, including how many reviewers screened each record and each report retrieved, whether they worked independently, and if applicable, details of automation tools used in the process.	Section 2.3, p. 4. Initial title/abstract screening was performed by L.J.; potentially eligible studies and final inclusion decisions were discussed with co-authors and resolved by consensus.
Data collection process	9	Specify the methods used to collect data from reports, including how many reviewers collected data from each report, whether they worked independently, any processes for obtaining or confirming data from study investigators, and if applicable, details of automation tools used in the process.	Section 2.3, p. 4-5. Data extraction was performed by L.J. and I.B.; disagreements were resolved by discussion
Data items	10a	List and define all outcomes for which data were sought. Specify whether all results that were compatible with each outcome domain in each study were sought (e.g. for all measures, time points, analyses), and if not, the methods used to decide which results to collect.	Sections 2.1 and 2.3, pp. 3–5. Table 1, p. 6. Outcomes are identified
	10b	List and define all other variables for which data were sought (e.g. participant and intervention characteristics, funding sources). Describe any assumptions made about any missing or unclear information.	Section 2.3, p. 5. Study design, follow-up, participant/tooth numbers, intervention, assessment methods and outcomes are listed
Study risk of bias assessment	11	Specify the methods used to assess risk of bias in the included studies, including details of the tool(s) used, how many reviewers assessed each study and whether they worked independently, and if applicable, details of automation tools used in the process.	Sections 2.3–2.4, pp. 4–5. RoB 1 tool, domains, assessors (L.J. and I.B.) and disagreement resolution are reported
Effect measures	12	Specify for each outcome the effect measure(s) (e.g. risk ratio, mean difference) used in the synthesis or presentation of results.	Not applicable as a meta-analysis was not performed

Section and Topic	Item #	Checklist item	Location where item is reported
Synthesis methods	13a	Describe the processes used to decide which studies were eligible for each synthesis (e.g. tabulating the study intervention characteristics and comparing against the planned groups for each synthesis (item #5)).	Sections 2.2–2.4, pp. 4–5. Eligibility and methodological-quality criteria determine inclusion in the qualitative synthesis
	13b	Describe any methods required to prepare the data for presentation or synthesis, such as handling of missing summary statistics, or data conversions.	Not reported.
	13c	Describe any methods used to tabulate or visually display results of individual studies and syntheses.	Not reported as a synthesis method. Results are displayed in Table 1 and Figures 1–2.
	13d	Describe any methods used to synthesize results and provide a rationale for the choice(s). If meta-analysis was performed, describe the model(s), method(s) to identify the presence and extent of statistical heterogeneity, and software package(s) used.	Partially reported: Abstract, p. 1 states that narrative synthesis was used because of substantial clinical heterogeneity
	13e	Describe any methods used to explore possible causes of heterogeneity among study results (e.g. subgroup analysis, meta-regression).	Not reported; no formal method for exploring causes of heterogeneity is described.
	13f	Describe any sensitivity analyses conducted to assess robustness of the synthesized results.	Not performed/reported.
Reporting bias assessment	14	Describe any methods used to assess risk of bias due to missing results in a synthesis (arising from reporting biases).	Not performed/reported.
Certainty assessment	15	Describe any methods used to assess certainty (or confidence) in the body of evidence for an outcome.	Not performed; Discussion, p. 12 explicitly states that no formal GRADE assessment was performed.
**RESULTS**			
Study selection	16a	Describe the results of the search and selection process, from the number of records identified in the search to the number of studies included in the review, ideally using a flow diagram.	Results, p. 6, and Figure 1, p. 3.
	16b	Cite studies that might appear to meet the inclusion criteria, but which were excluded, and explain why they were excluded.	Section 2.3, p. 4; Results, p. 6; Table S1 and Figure S1. Seven studies excluded on the predefined risk-of-bias threshold are cited as refs. [12–18], and reasons for exclusion are reported.
Study characteristics	17	Cite each included study and present its characteristics.	Table 1, pp. 6; Each included study is cited and its main characteristics are presented.
Risk of bias in studies	18	Present assessments of risk of bias for each included study.	Section 3.1, pp. 7–8, and Figure 2, p. 8. Risk-of-bias assessments for the nine included studies are presented.
Results of individual studies	19	For all outcomes, present, for each study: (a) summary statistics for each group (where appropriate) and (b) an effect estimate and its precision (e.g. confidence/credible interval), ideally using structured tables or plots.	Sections 3.2–3.8, pp. 8–10. Outcome results are described
Results of syntheses	20a	For each synthesis, briefly summarise the characteristics and risk of bias among contributing studies.	Results, pp. 6–10; Table 1 and Figure 2. Characteristics and risk of bias of contributing studies are summarized
	20b	Present results of all statistical syntheses conducted. If meta-analysis was done, present for each the summary estimate and its precision (e.g. confidence/credible interval) and measures of statistical heterogeneity. If comparing groups, describe the direction of the effect.	Not applicable; no statistical synthesis/meta-analysis was performed.

PRISMA

PRISMA 2020 Checklist

Section and Topic	Item #	Checklist item	Location where item is reported
	20c	Present results of all investigations of possible causes of heterogeneity among study results.	Not applicable/not performed; heterogeneity is discussed qualitatively in Discussion, pp. 11–12, but no formal investigation is reported.
	20d	Present results of all sensitivity analyses conducted to assess the robustness of the synthesized results.	Not performed/reported.
Reporting biases	21	Present assessments of risk of bias due to missing results (arising from reporting biases) for each synthesis assessed.	Not performed/reported.
Certainty of evidence	22	Present assessments of certainty (or confidence) in the body of evidence for each outcome assessed.	Not performed; Discussion, p. 12 states that no formal GRADE assessment was performed.
**DISCUSSION**			
Discussion	23a	Provide a general interpretation of the results in the context of other evidence.	Discussion, pp. 10–12. Results are interpreted in the context of previous evidence.
	23b	Discuss any limitations of the evidence included in the review.	Discussion, pp. 10–12, including methodological quality and heterogeneity of the evidence.
	23c	Discuss any limitations of the review processes used.	Discussion, p. 12: no prospective registration, no formal GRADE assessment, author-defined risk-of-bias exclusion threshold, and heterogeneity are identified as limitations.
	23d	Discuss implications of the results for practice, policy, and future research.	Discussion, pp. 11–12, and Conclusions, pp. 12–13. Implications for clinical interpretation and future research, including the need for standardized protocols and further high-quality RCTs, are discussed.
**OTHER INFORMATION**			
Registration and protocol	24a	Provide registration information for the review, including register name and registration number, or state that the review was not registered.	Section 2.1, p. 3: the review was not prospectively registered.
	24b	Indicate where the review protocol can be accessed, or state that a protocol was not prepared.	Section 2.1, p. 3: no formal review protocol was prepared.
	24c	Describe and explain any amendments to information provided at registration or in the protocol.	Not applicable; no registration or formal protocol was prepared.
Support	25	Describe sources of financial or non-financial support for the review, and the role of the funders or sponsors in the review.	Funding, p. 13: “This research received no external funding.”
Competing interests	26	Declare any competing interests of review authors.	Conflicts of Interest, p. 13: authors declare no conflicts of interest.

PRISMA 2020 Checklist

Section and Topic	Item #	Checklist item	Location where item is reported
Availability of data, code and other materials	27	Report which of the following are publicly available and where they can be found: template data collection forms; data extracted from included studies; data used for all analyses; analytic code; any other materials used in the review.	Data Availability Statement, p. 13. No new data were created or analyzed and data sharing is stated as not applicable; availability of extraction forms, extracted data, or analytic code is not otherwise reported.

From: Page MJ, McKenzie JE, Bossuyt PM, Boutron I, Hoffmann TC, Mulrow CD, et al. The PRISMA 2020 statement: an updated guideline for reporting systematic reviews. BMJ 2021;372:n71. doi: 10.1136/bmj.n71. This work is licensed under CC BY 4.0. To view a copy of this license, visit https://creativecommons.org/licenses/by/4.0/
